# Supplementary material for: A localized PCR inhibitor in a porcelain crab suggests a protective role
Source: PeerJ. 2014 Dec 4;2:e689. doi: 10.7717/peerj.689 (PMC4260131; doi:10.7717/peerj.689)
Supplement: Table S1 — PCR conditions for each primer used. Temperatures are in °C. Steps 2–5 were performed for 35 cycles. [file peerj-02-689-s001.docx]

| **Times** | **16s Temp** | **18s Temp** | **28s Temp** |
| --- | --- | --- | --- |
| 5 min | 94 | 94 | 94 |
| 30 sec | 94 | 94 | 94 |
| 30 sec | 52 | 48 | 56 |
| 1 min | 72 | 72 | 72 |
| 7 min | 72 | 72 | 72 |
| Hold | 4 | 4 | 4 |
