# Supplementary figures and images for: A localized PCR inhibitor in a porcelain crab suggests a protective role
Source: PeerJ. 2014 Dec 4;2:e689. doi: 10.7717/peerj.689 (PMC4260131; doi:10.7717/peerj.689)

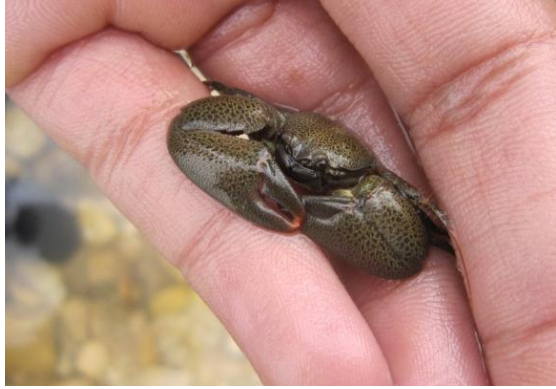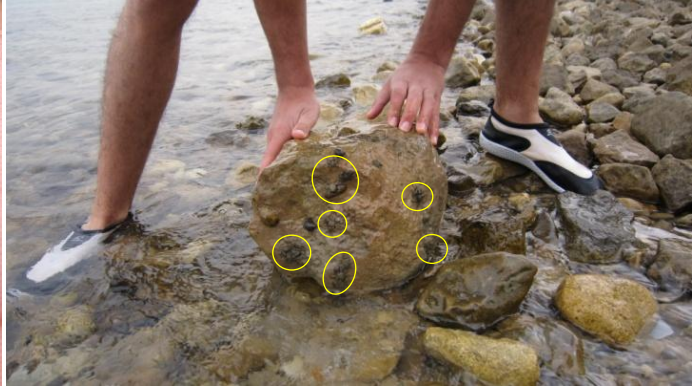

Supplement: Figure S1 — A single Petrolisthes rufescens on an investigator’s hand (left), and several individuals (ca. 15) from the underside of a rock (right, inside the yellow circles). Crabs were abundant among tan and dark rocks that were exposed at low tide and heated by full sunlight for hours. The surrounding coastal environment is the characteristic desert at this region of the Gulf of Suez (photos by E Cruz-Rivera). [file peerj-02-689-s002.pdf]

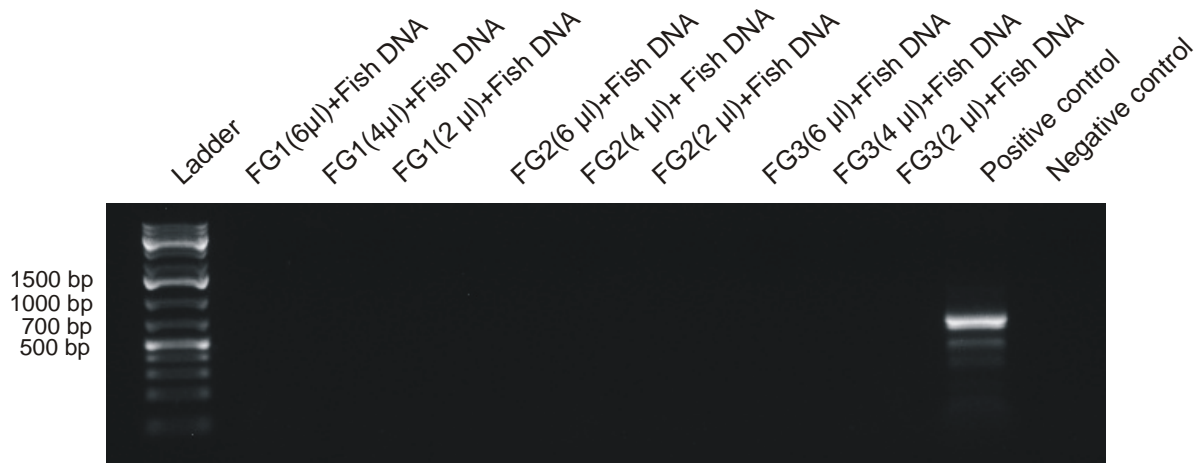

Supplement: Figure S2 — Amplification of fish DNA using 28s primers in the presence or absence of foregut (FG) extracts. Three amounts of extracted foregut aliquots from three different crabs were added to the PCR reactions, but these were not standardized by mass using NanoDrop (see Materials and Methods) and were done strictly by volume. The positive control contained only fish DNA (2 ng) for amplification and the negative control (to assess potential contamination with foreign DNA) contained no crab or fish DNA. Empty lanes in the gel are not labeled. [file peerj-02-689-s003.pdf]

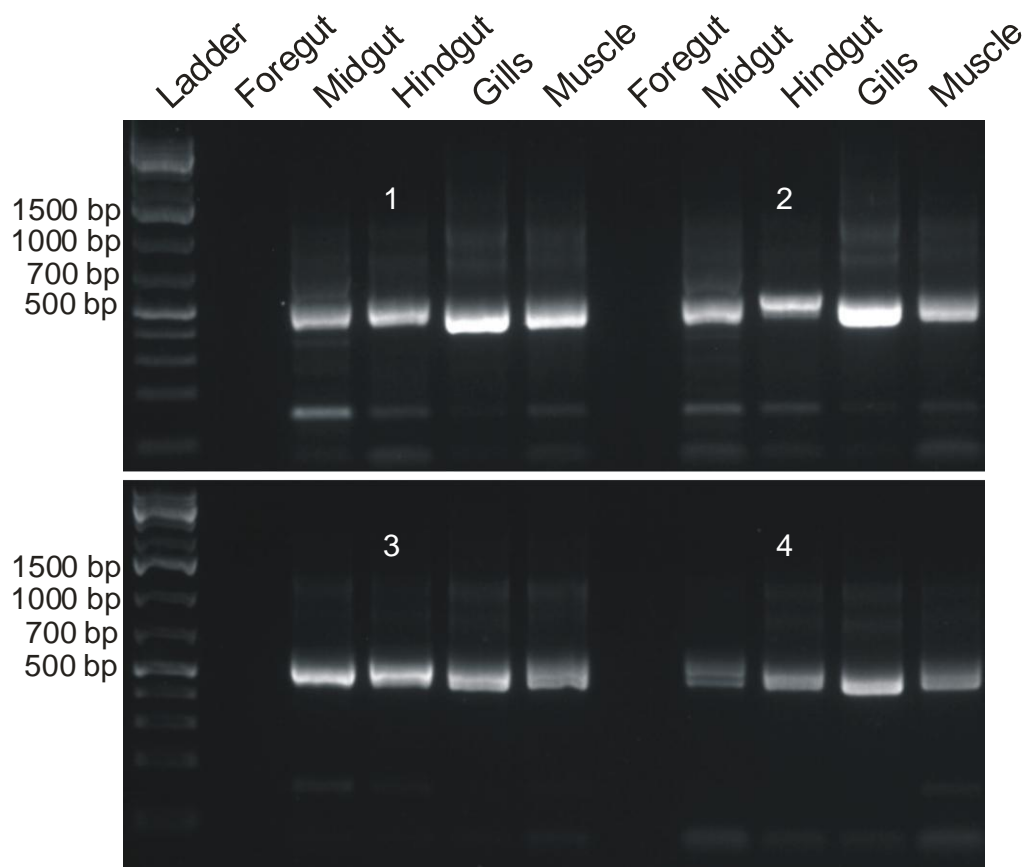

Supplement: Figure S3 — Two separate gels showing amplification of bacterial DNA, from four different crabs, using universal 16s primers. All parts in all crabs, except the foreguts, yielded positive amplification products. These (among other) preliminary observations strongly suggested the presence of a PCR inhibitor restricted to the foregut of P. rufescens. DNA amounts ranged between 0.52 and 47.63 per PCR reaction in these experiments. [file peerj-02-689-s004.pdf]
