# Supplementary material for: A localized PCR inhibitor in a porcelain crab suggests a protective role
Source: PeerJ. 2014 Dec 4;2:e689. doi: 10.7717/peerj.689 (PMC4260131; doi:10.7717/peerj.689)
Supplement: Supplemental Information 5 — DNA yield of extracted crab parts. [file peerj-02-689-s005.pdf]

Data from Table 1: DNA yield of extracted crab parts

M. Dessouki, A. Ouf

| Crab part | DNA extracted ng/microl |
|---|---|
| Foregut | 3.123 |
| Midgut | 9.527 |
| Hindgut | 0.514 |
| Gills | 4.442 |
| Foregut | 0.685 |
| Midgut | 0.132 |
| Hindgut | 0.115 |
| Gills | 0.198 |
| Foregut | 0.156 |
| Midgut | 0.168 |
| Hindgut | 1.019 |
| Gills | 1.405 |
| Foregut | 0.236 |
| Midgut | 0.104 |
| Hindgut | 0.204 |
| Gills | 0.110 |
| Foregut | 0.248 |
| Midgut | 0.003 |
| Hindgut | 0.332 |
| Gills | 0.156 |
| Foregut | 0.166 |
| Midgut | 1.578 |
| Hindgut | 0.737 |
| Gills | 0.128 |
