# Supplementary material for: A localized PCR inhibitor in a porcelain crab suggests a protective role
Source: PeerJ. 2014 Dec 4;2:e689. doi: 10.7717/peerj.689 (PMC4260131; doi:10.7717/peerj.689)
Supplement: Supplemental Information 6 — Absorbances for spectrophotometric melanin test. [file peerj-02-689-s006.pdf]

Absorbance for potential melanins in crab/fish tissues

Absorbance at 320 nm

05-Feb-13

Mahmoud Desouki

Sample	Abs. 1	Abs. 2	Avg. Absorbance
Foregut	0	0.002	0.001
Foregut	0.004	0.006	0.005
Foregut	-0.007	0.007	0
Foregut	-0.015	-0.012	-0.0135
Midgut	-0.007	-0.004	-0.0055
Midgut	-0.007	-0.007	-0.007
Midgut	0.003	0.002	0.0025
Midgut	0.018	0.001	0.0095
Hindgut	-0.001	-0.002	-0.0015
Hindgut	0	0.002	0.001
Hindgut	0.015	0.011	0.013
Hindgut	0.006	0.012	0.009
Gills	0.004	0.012	0.008
Gills	0.023	-0.002	0.0105
Gills	0.009	0.007	0.008
Gills	0.004	-0.001	0.0015
Muscle	-0.002	0.003	0.0005
Muscle	-0.005	-0.004	-0.0045
Muscle	0.1	0.048	0.074
Muscle	0.02	0.014	0.017
Fish muscle	-0.008	-0.002	-0.005
Fish muscle	-0.006	0.001	-0.0025
Fish muscle	-0.001	-0.007	-0.004
Fish muscle	0.006	-0.007	-0.0005
